# Suppuration without Micro-Organisms

**Published:** 1882-04

**Authors:** 


### SUPPURATION WITHOUT MICRO-ORGANISMS.

Dr. Uskoff, of Cronstadt, records in *Virchow's Archives* a series of interesting experiments, which appear to prove that suppuration can occur independently of micro-organisms. Small quantities of distilled water, milk, oil, pus, turpentine, etc., were injected subcutaneously into the cellular tissues of dogs, the tissues being examined microscopically a few days later. Bland fluids, like water and milk, if injected in small quantities, fifteen to thirty minims, caused no suppuration ; large abscesses formed if the quantity injected amounted to a drachm or more. The injection of turpentine produced, in most cases, large abscesses, the pus being entirely free from micro-organisms. Dr. Uskoff concludes that, although in many cases suppuration is caused by micro-organisms, yet it may occur independently of them, being caused by chemical irritation, as when turpentine is injected, or to damage of tissues, as when water is forcibly injected.—*Med. Herald.*

